# The Cellular and Molecular Landscape of Synchronous Pediatric Sialoblastoma and Hepatoblastoma

**DOI:** 10.3389/fonc.2022.893206

**Published:** 2022-07-04

**Authors:** Ran Yang, Yong Zhan, Yi Li, Shu-Yang Dai, Shi-Wei He, Chun-Jing Ye, Ling-Du Meng, De-Qian Chen, Chen-Bin Dong, Lian Chen, Gong Chen, Kui-Ran Dong, Kai Li, Shan Zheng, Jun Li, Wei Yao, Rui Dong

**Affiliations:** ^1^ Department of Pediatric Surgery, Children’s Hospital of Fudan University, Shanghai Key Laboratory of Birth Defect, Shanghai, China; ^2^ Department of Pathology, Children’s Hospital of Fudan University, Shanghai, China

**Keywords:** hepatoblastoma, sialoblastoma, single cell analysis, embryonal neoplasm, genetic predisposition to cancer

## Abstract

Sialoblastoma (SBL) is an infrequent embryonal malignant tumor originating from the salivary gland, resembling primitive salivary gland anlage, whereas hepatoblastoma (HB) is the most common pediatric liver malignancy. The simultaneous occurrence of both tumors is extremely rare. Here we reported a case of a 6-month-old infant diagnosed with synchronous SBL and HB. The patient received neoadjuvant chemotherapy followed by surgical resection. Fresh tissues of both tumors were collected before and after chemotherapy, which were further profiled by whole exome sequencing (WES) and single-cell RNA sequencing (scRNA-seq). WES analysis revealed potential somatic driver mutation *PIK3CA* p.Glu454Lys for SBL and canonical mutation *CTNNB1* p.Ser45Pro for HB. No shared somatic variants or common copy number alterations were found between SBL and HB primary tumor samples. Though scRNA-seq, single-cell atlases were constructed for both tumors. SBL may recapitulate a pre-acinar stage in the development of salivary gland, including basaloid, duct-like, myoepithelial-like, and cycling phenotypes. In the meantime, HB was composed of tumor cells resembling different stages of the liver, including hepatocyte-like, hepatic progenitor-like, and hepatoblast-like cells. After chemotherapy, both tumors were induced into a more mature phenotype. In terms of transcriptional signatures, SBL and HB showed enhanced expression of epithelial markers *KRT8, KRT18*, and essential embryo development genes *SDC1, MDK*, indicating the disruption of normal embryo epithelium development. Finally, heterozygous deleterious germline mutation *BLM* and *FANCI* were identified which could predispose the patient to higher cancer risk. It partially explained the reason for the co-occurrence of SBL and HB. Taken together, we provided valuable resources for deciphering cellular heterogeneity and adaptive change of tumor cells after chemotherapy for synchronous SBL and HB, providing insights into the mechanisms leading to synchronous pediatric tumors.

## Introduction

Sialoblastoma (SBL) is an extremely rare embryonal epithelial malignancy derived from the salivary gland, which recapitulates the structure of primitive salivary gland. Only about 80 cases have been reported in the literature worldwide. SBL is usually diagnosed at birth or infancy with identifiable cheek mass enlargement. It predominantly occurs in the major salivary gland, with parotid gland more common than submandibular gland (1). As for histological features, the tumor is composed of solid nests or islands of basaloid cells with scant cytoplasm at the periphery and central ductal-like cells. It is separated by fibrous tissue and myoepithelial-like cells (2). Immunohistochemistry (IHC) results showed tumor cells were positive for epithelial markers, e.g., *EPCAM*, *CK7*, and *CK8*. Vimentins and SMA were positive in fibrous stroma with myoepithelial cells. Ki-67 proliferation index varies between patients, with a high index indicating unfavorable outcomes (3, 4). Out of its rarity, few studies have revealed transcriptional features and pathogenesis of SBL. Consequently, there is no agreement on the management of this disease. Surgical resection with negative margins plays a core role, while radiotherapy or neoadjuvant chemotherapies protocols from other solid tumors have also been applied (5).

Unlike the rarity of SBL, hepatoblastoma (HB) is the most common primary liver tumor of childhood. The incidence of HB is 1.5 cases per million each year (6). HB is proposed to be derived from hepatic precursor cells and is morphologically similar to immature fetal hepatocytes. The mutation or deletion of ß-catenin encoding gene (*CTNNB1*) exon is the most frequently detected gene in HB, resulting in the activation of WNT signally pathways (7). Cairo et al. classified HB as C1 and C2 subtype at the transcriptome level, with C2 presenting a highly proliferative phenotype and signifying poor prognosis (8). This classification was further improved that C2a and C2b subtypes were defined based on *VIM* expression (9). The current standard of care is comprised of neoadjuvant or adjuvant chemotherapy regimens based on PRETEXT staging system, combined with surgical resection or liver transplantation. Though many studies have investigated genomic and transcriptomic features of HB, few have elucidated intratumoral heterogeneity and tumor evolution after chemotherapy.

Synchronous tumors are defined as cases in which more than one primary tumor in a single patient is identified at initial presentation, which is extremely rare, especially for children. The most common reported pediatric synchronous tumor was Wilms tumor and neuroblastoma. The majority of these patients were also diagnosed with Fanconi Anemia (FA), an autosomal recessive inherited syndrome characterized by congenital defects, aplastic anemia, and a high likelihood for cancer (10, 11). As for simultaneous SBL and HB, only 5 cases have been previously reported without in-depth investigation of potential mechanisms (12–16). Underlying molecular and cellular features of simultaneous SBL and HB were poorly understood.

**Figure 1:**
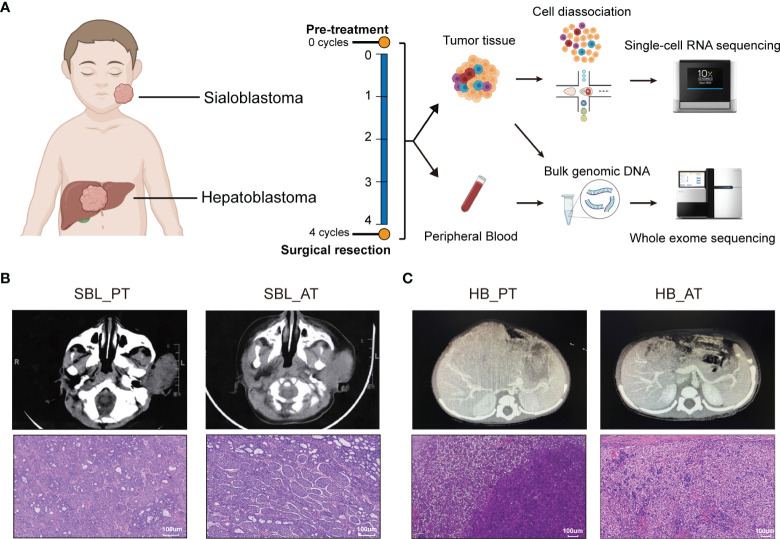
The occurrence of two synchronous pediatric tumors. **(A)** Diagrams of the study design and sample workflow (Created with BioRender.com). Fresh tumor specimens were collected at the timepoint of surgical biopsy and tumor resection. **(B)** CT images and H&E staining of SBL before and after neoadjuvant chemotherapy. **(C)** CT images and H&E staining of HB before and after neoadjuvant chemotherapy. CT, Computed tomography; H&E, hematoxylin and eosin; SBL, sialoblastoma; HB, hepatoblastoma; PT, primary tumor; AT, tumor after treatment.

In the current study, we used whole exome sequencing (WES) and single-cell RNA sequencing (scRNA-seq) to profile the genome and transcriptome of SBL and HB tumor tissues from the same patient before and after chemotherapy (**Figure 1A**). We have built a cellular atlas and characterized the genomic as well as transcriptomic profiles of synchronous SBL and HB, elucidating how they evolve after treatment. Our study highlighted the intratumoral heterogeneity of both tumors and each subtype displayed distinct signatures reminiscent of different developmental stages of normal cells. Furthermore, we have found heterozygous deleterious germline mutations that might contribute to the co-occurrence of the two embryonal tumors. These results shed light on the cellular and molecular features of synchronous tumors and their potential mechanisms.

## Materials and Methods

### Patient and Tumor Samples

This study was approved by the Institutional Review Board of Children’s Hospital of Fudan University. Informed consent was obtained from the legal guardians of the patients. The diagnosis of sialoblastoma and hepatoblastoma was confirmed by two pathologists according to the hematoxylin and eosin (H&E)-stained sections. Samples of tumor tissue were collected during the biopsy and resection surgery.

### Whole-Exome Sequencing (WES) DNA Isolation, Library Construction and Sequencing

Tumor tissues collected at diagnosis and surgical resection were stored as fresh frozen tissue. Peripheral blood collected from the patient was used as a matched control for tumor samples. DNA was prepared using the HiPure Universal DNA Kit (Angen Biotech) according to the manufacturer’s instructions. The library was constructed using the Vazyme TruePrep™ DNA Library Prep Kit V2 for Illumina (Vazyme Biotech). The SeqCap EZ Exome Probes v3.0 (Roche) was used to capture exome regions. The libraries were further sequenced on NovaSeq 6000 (Illumina).

### SNV and Indel Mutation Identification

Reads were aligned to the human reference genome (GRCh38) using Burrows–Wheeler Aligner (0.7.15). We used Genome Analysis Toolkit (GATK version 4.2.0) to process BAM files, including sorting bam files, marking duplicates, and local realignment around high confidence insertion and deletions. Single nucleotide variants (SNVs) and short insertions/deletions (indels) for each sample were called using GATK HaplotypeCaller (https://gatk.broadinstitute.org/hc/en-us/articles/360035535932-Germline-short-variant-discovery-SNPs-Indels-), followed by joint genotyping using GATK GenotypeGVCFs. Hard-filtering with VariantFiltration was undertaken independently for SNPs and indels. Candidate germline variants were searched in the COSMIC database (the Catalogue Of Somatic Mutations In Cancer; http://cancer.sanger.ac.uk/cosmic) and ClinVar (https://www.ncbi.nlm.nih.gov/clinvar), and their potential effect on protein function was predicted with CADD, SIFT and PolyPhen. Germline variants with CADD PHRED> 20, SIFT deleterious, PolyPhen probably/possibly damaging and gnomAD exome frequency <0.002 were considered as potential pathogenic germline variants. Somatic SNVs and indels in tumor samples were called using the MuTect2 workflow (https://gatk.broadinstitute.org/hc/en-us/articles/360035894731-Somatic-short-variant-discovery-SNVs-Indels-). Variants from germline resources of population data, e.g., gnomAD, 1000 Genomes were also removed. All variants were annotated using ANNOVAR (http://www.openbioinformatics.org/annovar/. Variants with depth were removed from subsequent analyses if the position was <10× in normal and tumor samples. The R package “maftools” (http://bioconductor.org/packages/maftools/) was used for the visualization of somatic mutation data.

### Copy Number Variation Analysis

Copy Number Variation (CNV) calling was done using the GATK (v 4.2.0) somatic CNV calling pipeline (https://gatk.broadinstitute.org/hc/en-us/articles/360035531092–How-to-part-I-Sensitively-detect-copy-ratio-alterations-and-allelic-segments#ref2). The peripheral blood sample from the patient was used as a control to identify tumor-specific genomic alterations. Called segments were further annotated with gene-based and region-based information using Annovar.

### Validation of Variant Using Sanger Sequencing

Candidate germline variants identified through WES were validated using Sanger sequencing. Whole blood DNA was isolated according to the manufacturer’s protocol with QIAamp DNA Mini Kit (QIAGEN, Cat.51306) and quantified by NnodropOne(Thermo fisher). Premiers were designed as follows.

**Table:** 

BLM-F-F	GATTCCAGCTACATATCTGACAGGTGA
BLM-R-R	TGCATGCATGATCTGGGATTT
GOLGA5-F-F	TTGTTTGAACCTTGAGATGCATTGT
GOLGA5-R-R	TCACAGAAACAGAATTTTCTGAAATGC
USP44-F-F	AGCAAATGTAAGTCATTTACTAGCCA
USP44-R-R	GATCTGTCTAACTTGACTAGCTCCTA
USH2A-F-F	TAAAGAAACCAACATCTGTGGCTAA
USH2A-R-R	CTGCCTTGGTCAAGAGCTCAAA
EPS15-F-F	CTGCCAAGCAACACAGCATTTTA
EPS15-R-R	TCCAGTTAGTAAGATTGCAGATCACT
FANCI-F-F	ACCTGTTCGTTTTTCCTATTTACCTGT
FANCI-R-R	TCCTTCCCTCAACAAATTACAAACCC

Sequencing was performed with BigDye™ Terminator v3.1 cycle sequencing kit (Applied Biosystems. cat.4337457) on ABI 3730XL genetic analyzer (Applied Biosystems). Sequencing chromatograms were analyzed by Chromas (http://technelysium.com.au/wp/chromas/).

### scRNA-Seq Sample Preparation

Samples were processed within an hour after surgery. Each tissue was cut into 1×1×1 mm fragments and minced on a plate. Then the specimens were enzymatically digested with collagenase IV (Gibco) and DNase I (Sigma) for 30 min at 37°C with agitation. After digestion, specimens were sieved through a 70-μm cell strainer, centrifuged for 5 minutes at 400 g, suspended in Dulbecco’s modified Eagle’s medium (DMEM, Gibco) with 10% fetal bovine serum albumin (FBS), and centrifuged through Lympholyte-H separation (CL5020; Cedarlane) to remove RBCs for 20min, 800 g. Pelleted cells were then re-suspended in DMEM with 10% FBS and assessed for scRNA-seq.

### Generation of scRNA-Seq Data

An estimated 8,000 to 10,000 cells were targeted for capture per sample. Then, cell suspensions of each sample were run in the Chromium Controller with appropriate reagents to generate single-cell gel bead-in-emulsions (GEMs) for cell barcoding. The libraries were then pooled and sequenced on NovaSeq 6000 (Illumina) at a depth of approximately 400M reads per sample. Raw sequencing data were converted to FASTQ files with Illumina bcl2fastq, version 2.19.1, and aligned to the human genome reference sequence (GRCH38). The CellRanger (10X Genomics, 3.0.1 version) analysis pipeline was used to sample demultiplexing, barcode processing and single-cell 3′ gene counting to generate a digital gene-cell matrix from this data.

### Quality Control and Unsupervised Clustering

Scrublet Package was used to remove cell doublets (17). The Pegasus package (18)was used to process and analyze the gene expression matrix. To filter out low-quality cells, we removed cells with less than 200 genes or more than 6500 genes detected. Cells with over 20% genes derived from the mitochondrial genome were also removed. We excluded nuclear mitochondrial genes and ribosomal genes from the following analysis. In addition, we also removed cells expressing two cell-type markers. In SBL data, one cluster of cells highly expressed T/NK cell markers (*NKG7, GNLY, GMZA*) and keratins *(KRT19, KRT14)*, which might be T/NK cells contaminated by tumor RNA during the process of sample preparation and library construction. These cells were excluded from the following analysis.

After quality control, the robust genes were identified by *pg.identify_ robust_genes* function. Then data were log normalized with the function *pg.log_norm*. Highly variable genes were identified and analyzed by *pg.highly_variable_features* function. Principal component analysis was performed with the *pg.pca* function. The batch correction was achieved by pg.run_harmony function. Clusters were identified by *pg.neighbors*, *pg.louvain* function and visualized by t-distributed stochastic neighbor embedding (t-SNE) and uniform manifold approximation and projection (UMAP) using *pg.tsne* and *pg.umap* function, respectively. DEGs of a given cell type compared with all other cell types were determined with the *pg.de_analysis* and *pg.markers* function from the Pegasus package by Mann-Whitney U (MWU) test.

### InferCNV Analysis

We identified malignant tumor cells by inferring large-scale chromosomal copy-number variations (CNVs) in each cell (19). We used endothelial cells and fibroblasts in each dataset as reference cells. Since whole exome sequencing showed chr5q loss before and after chemotherapy in SBL, we extracted chr5q loss status and calculated chr5q loss score from ”map_metadata_from_infercnv. txt” in inferCNV results.

### Differentially Expressed Genes Analysis

The Deseq2 package (20) was used to perform the differentially expressed genes (DEGs) analysis between primary (PT) and post-chemotherapy (AT) tumor samples. The gene expression matrix was extracted from the Pegasus object. And the DEGs were screened by satisfying p-value <0.05, abs (fold change (FC))>1.5.

### Functional Enrichment Analysis

The Gene Ontology (GO) and Kyoto Encyclopedia of Genes and Genomes (KEGG) pathway analysis were performed to investigate the cell function status by using the R package, clusterProfiler (21). FDR p-value < 0.05 was used to distinguish significantly enriched terms. “enrichGO” and “enrichKEGG” are used. Gene set enrichment analysis (GSEA) was performed using MSigDB collections(https://www.gsea-msigdb.org/gsea/msigdb/index.jsp) through the function “GSEA” in clusterProfiler.

### Assessing Similarity Between Tumor Cell Subpopulations and Developmental Cell Types

We utilized murine salivary gland datasets GSE150327 as the reference dataset to evaluate the similarity between tumor cell subpopulations and developmental cell types (22). To increase the accuracy of cell classification, we simplified original cell definitions into embryo end bud, embryo duct, embryo basal duct, embryo myoepithelial, postnatal basal duct, postnatal duct, postnatal proacinar, postnatal acinar, and postnatal myoepithelial cells. R package biomaRt (v 2.46.3) was used to convert mouse to human gene names. R package Garnett for Monocle 3 (v 0.2.17) (23)was used to generate cell type classifiers in the maker-free mode with parameters: max_training_samples = 2000, num_unknown = 5. We applied the function classifiy_cells to sialoblastoma malignant cell datasets using the trained classifiers with the following non-default parameters: cluster_extend = TRUE, rank_prob_ratio=1.2, cluster_extend_max_frac_unknown=0.2.

### SCENIC Analysis

pySCENIC was applied to run the SCENIC analysis (24). The analysis is composed of pre-processing data, network inference, module generation, motif enrichment, TF-regulon prediction and cellular enrichment. We calculated Regulon specificity score (RSS) for each metaprogram. The top 10 regulons with the highest RSS were used for plotting heatmap of regulon activities. All tumor cells were further clustered by regulon activities.

### Determination of Cell Developmental Potential

The R package CytoTRACE (0.3.3) was used for investigating the developmental potential of hepatoblastoma tumor subpopulations. The top 200 genes correlated with CytoTRACE values were chosen as markers for immaturity, respectively. Gene set enrichment analysis was further performed using MSigDB (https://www.gsea-msigdb.org/gsea/msigdb/index.jsp).

## Results

### Clinical Characteristics of Patient With Synchronous SBL and HB

A 6-month-old infant with synchronous growing left-sided firm cheek mass and abdominal mass was referred to our institution. Computed tomography (CT) scan showed the existence of a soft tissue tumor in the right parotid gland (about 66×61×43 mm^3^) as well as a heterogeneous lesion affecting the left and right lobes of the liver (about 91×85×85 mm^3^). Whole-body bone scan showed no systemic metastasis. Lab tests demonstrated elevated levels of blood tumor markers, including alpha-fetoprotein (AFP), carcinoembryonic antigen (CEA) and neuron-specific enolase (NSE) (**Table 1**). The patient was then subjected to excisional biopsy and diagnosed as SBL with HB based on pathological examination results (**Figures 1B, C** and **Supplementary Figure S1**). No other congenital abnormalities were identified. After receiving four cycles of neoadjuvant chemotherapy adopting an intermediate-risk neuroblastoma regimen (carboplatin, cyclophosphamide/etoposide, and doxorubicin), the volume of both tumors showed an obvious decrease (post-treatment size: 60×59×42 mm^3^ for SBL, 50×30×15 mm^3^ for HB). Though still higher than normal, the level of blood tumor markers decreased. Segmental liver resection (4b, 5, 6) was performed at the age of 10 months, achieving complete resection of the tumor. Surgical resection of the whole facial mass and partial parotid sialoadenectomy was performed simultaneously. The buccal branch of the facial nerve was infiltrated and thus not preserved. Then the patient received another four courses of chemotherapy. Clinical characteristics and immunohistochemical (IHC) features of SBL and HB at the timepoint of biopsy and surgical resection were summarized in **Table 1**. No signs of tumor recurrence or metastasis were observed during the 32-month follow-up after surgery.

**Table 1:** Clinical information and immunohistochemical results.

Timepoint	Age	Tumor Site	Tumor size*(mm^3^)	Tumor markers (ng/ml)	H&E staining	IHC staining		
						Markers	Ki67+(%)	
**Biopsy**	6M14D	Left Parotid gland	66×61×43	AFP 121,000.00 ↑; CEA 5.79 ↑; NSE 30.30 ↑; FERR 169.70	Nests of basaloid cells with cribriform pattern and peripheral palisading;	CK (+), p63(+/-)	25%
		Right and left lobe of liver	91×85×85		Hepatoblastoma with mixed fetal and embryonal phenotype	Hepo (+), AFP (+), CK (+), SALL4(+/-), p63(-), BESP (-)	20-30%	
**Surgical** **resection**	10M20D	Left Parotid gland	60×59×42	AFP 3888.00↑ ; CEA 5.23↑ ; NSE 18.78↑ FERR 126 ;	Nests of basaloid cells with cribriform pattern and peripheral palisading; More ductal structures;	CK18(+), CK(+/-), S100(+), CK7(+), CK19(+), SMA(+), P63(+), WT-1(+/-), AFP(-), Calponin(+)	20%	
		Liver (segment 4b, 5, 6)	50×30×15		Tumor cells differentiating towards hepatocytes with partial tumor necrosis	Hepo(+), AFP(+/-), PLAP(-), CK7(-), β-catenin (-), CK(+/-), VIM(-), BESP(+), INI-1(+), P53(-)	5-10%	

*Estimated through CT image; ↑ Elevated level of tumor markers; H&E, Haemotoxylin and Eosin; IHC, Immunohistochemical.

### Uncovering Genomic and Cellular Landscape of SBL

In order to characterize the cellular landscape of SBL and HB, and tumor remodeling after chemotherapy, we collected fresh specimens before and after chemotherapy, encoded as primary tumor (PT) and tumor after treatment (AT), respectively, which were further subjected to scRNA-seq and WES (**Figure 1A**; **Supplementary Tables 1, 2**).

**Figure 2:**
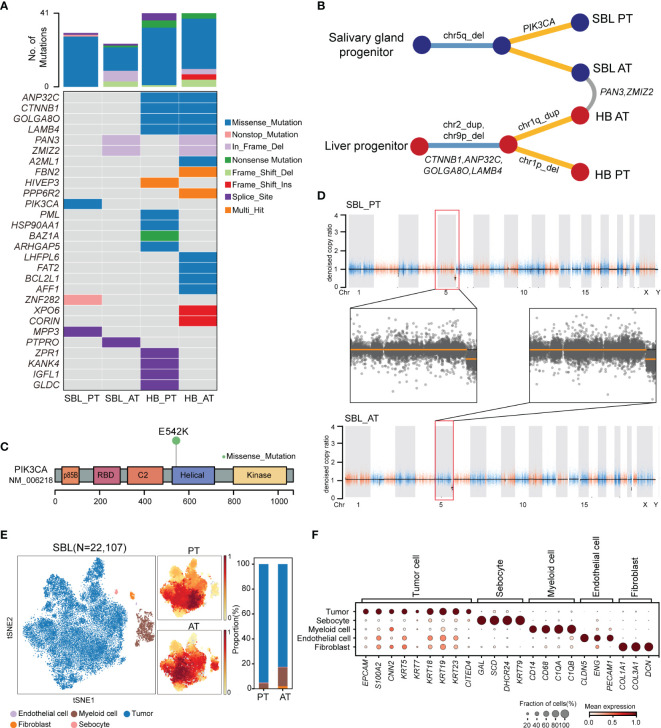
The genomic and transcriptomic landscape of SBL. **(A)** Oncoplot outlining the distribution of mutations across 4 tumor samples. The color indicated different variant types. **(B)** Phylogenetic tree diagram showing key somatic mutations as well as shared mutated genes and copy number alterations. **(C)** Lollipop plot showing the hot spot mutation of *PI3KCA* p.E542K. Boxes represent functional domains. p85B, p85 binding domain; RBD, Ras binding domain. **(D)** Scatter plots showing copy number alterations in SBL PT and AT samples. **(E)** tSNE visualization of 22,107 cells from SBL colored by cell types (left) and density of sample distribution(middle). The right panel shows the proportions of different cell types in SBL PT and AT samples. tSNE, t-distributed stochastic neighbor embedding. **(F)** Dot plot for expression of cell-type-specific markers in SBL. The color indicated the scaled mean expression of marker genes, and the size indicated the proportion of cells expressing marker genes. SBL, sialoblastoma; HB, hepatoblastoma; PT, primary tumor; AT, tumor after treatment.

For SBL, WES analysis revealed that PT and AT harbored 30 and 24 non-silent coding mutations, respectively (**Figure 2A**, **Supplementary Table 3**). No common variants were found between two samples, possibly due to tumor heterogeneity or tumor evolution after chemotherapy. A hotspot gain-of-function mutation *PIK3CA* E542K was identified in the PT sample, located in the helical domain of PIK3CA (**Figure 2C**). This variant has been proven to be oncogenic and associated with tumor proliferation and progression in several other tumors, including salivary duct carcinoma, breast and cervical cancer (25, 26). It may also play an essential role in the carcinogenesis of SBL. Mutated genes in PT and AT samples were enriched in similar pathways such as PI3K-Akt and Wnt signaling pathways (**Supplementary Figure S2A, B**). Although there were no common variants shared between T1 and T2, they showed consistent chromosome 5q34-5q35.3 loss (**Figures 2B, D**; **Supplementary Table S4**).

After analyzing its genomic landscape, we next turned to explore its transcriptional features at the single-cell level. After removing low-quality cells and potential doublets, 22,107 high-quality cells were proceeded to subsequent analysis. Following normalization of gene expression profiles and graph-based clustering on principal-component analysis (PCA) analysis, t-distributed stochastic neighbor embedding (tSNE) plots were used for visualization (**Figure 2E**). We identified 5 major cell types based on canonical markers, including epithelial cells (*KRT5, KRT14, KRT18, KRT19, S100A2*) *(* 27), myeloid cells (*CD14, CD68, C1QA, C1QB*), fibroblasts (C*OL1A1, COL3A1*), endothelial cells (*CLDN5, ENG, PECAM1*) and a cluster of sebocytes which highly expressed fatty-acid and sterol synthesis genes such as *SCD*, *SOAT1* and *DHCR24*, as well as high quantity cytokeratin gene *KRT79* (28) (**Figure 2F**).

In order to identify malignant cells from non-malignant cells, we first calculated the average score of sialoblastoma markers reported in previous studies (*KRT5, KRT14, KRT23*, *TP63*, *EPCAM*, *KRT7, KRT19, KRT18, ACTA2, CNN2, S100A2*), showing that epithelial cells have the highest tumor score (**Supplementary Figure S2C**). Next, we utilized inferred copy-number variation (CNV) to differentiate malignant cells from non-malignant ones (19). Consistent with WES results showing tumor cells had chromosome 5q loss before and after chemotherapy, inferred CNV analysis revealed that epithelial cells displayed significantly higher chromosome 5q loss scores than other cells (two-sided Wilcoxon test, pvalue<2.2e-16) (**Supplementary Figure S2D, E**). Therefore, epithelial cells were identified as malignant cells, constituting the major components of SBL tumor ecosystems.

### SBL May Resemble a Pre-Acinar Stage in the Development of Salivary Gland

**Figure 3:**
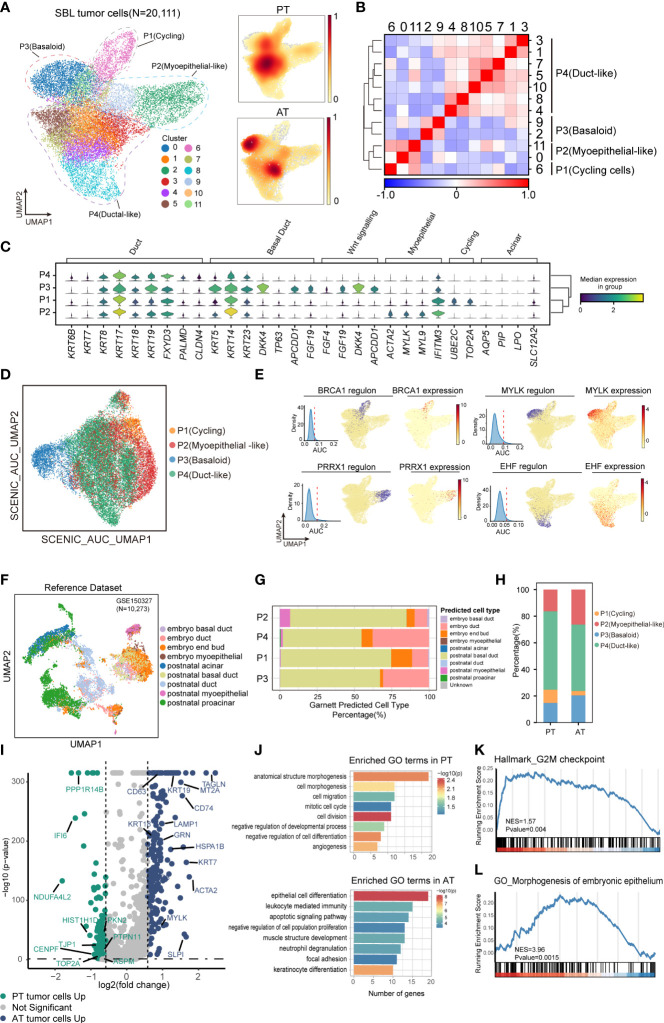
The intratumoral heterogeneity within SBL tumor cells. **(A)** UMAP visualization of 20,111 tumor cells from SBL colored by clusters (left) and density of sample distribution(right). UMAP, uniform manifold approximation and projection. **(B)** Heatmap displaying the Pearson correlation coefficients calculated between average gene expressions of tumor clusters. **(C)** Violin plots showing the expression distribution of selected genes involved in cell cycle genes, Wnt signaling pathway, and markers specific to duct, myoepithelial, basal and acinar cells in SBL tumor expression programs. **(D)** UMAP visualization of 20,111 tumor cells from SBL based on Scenic regulon AUC scores. AUC, area under the ROC Curve. **(E)** UMAP visualization of specific transcription factor expression and regulon activity. For four transcription factors: (left) histogram of AUC values, together with the chosen cutoff (red dashed line); (middle)the cells with AUC value over the cutoff value are shown in purple, where the regulon is considered active; (right) the expression of the transcription factor is shown. **(F)** UMAP visualization of murine embryo and postnatal salivary gland datasets curated from GSE150327.Colors indicated different cell types used for Garnett classifier training. **(G)** Bar plot indicating the percentage of tumor subtypes corresponding to 9 developing cell types. Tumor cells were assigned to different identities predicted by Garnett. **(H)** Bar plot indicating the percentage of tumor subtypes in SBL PT and AT samples. **(I)** Volcano plot showing differentially expressed genes between PT (green dots) and RT tumor cells (blue dots). The names of selected important genes are indicated in the plots. **(J)** Bar chart showing the enrichment of GO terms, based on top50 upregulated genes in PT or AT malignant cells. GO, gene ontology. **(K)** GSEA enrichment plot of expression signatures of Hallmark_G2M checkpoint in SBL PT malignant cells. **(L)** GSEA enrichment plot of expression signatures of GO_Morphogenesis of embryonic epithelium in SBL AT malignant cells. SBL, sialoblastoma; PT, primary tumor; AT, tumor after treatment.

Following identifying malignant populations, we further explored the transcriptional diversity of malignant cells. Following extracting and re-clustering all SBL tumor cells, we obtained a total of 12 clusters (**Figure 3A**). Based on gene expression pattern and transcriptional similarity, 12 clusters could be further converged into 4 different programs (**Figure 3B**; **Supplementary Figure S2F**, **Supplementary Table 5**).

P1 was enriched for cell cycle genes (e.g., *TOP2A, UBE2C, CKS2*), thus defined as cycling tumor cells. P2 was identified as myoepithelial-like cells by expressing muscle contraction and myofibril genes (e.g., *ACTA2, MYL, FHL2*), resembling myoepithelial cells in the salivary gland. P3 was potentially basaloid cells and expressed significantly higher canonical basal and progenitor cell markers, e.g., *KRT5, KRT14, KRT23* (29), suggesting an immature cell state (**Figure 3C**). Wnt signaling pathway inhibitors (e.g., *DKK4, APCDD1, NKD1*) and fibroblast growth factors (e.g., *FGF4, FGF19, FGF20*) were also tightly involved in its signatures. It has been revealed that the FGF signaling pathway could inhibit the Wnt pathway to repress ductal fate and help maintain the undifferentiated state of bud cells (30), which we supposed might be one of the mechanisms leading to the immature state of basaloid cells. P4 (duct-like cells) exhibited higher expression of common epithelial differentiation markers (e.g., *KRT6B*, *KRT7, KRT8, KRT18, KRT19*), and lower expression of basal or myoepithelial genes, demonstrating a more mature phenotype. Identification of divergent tumor phenotype was consistent with histology results, which revealed SBL was composed of solid nests of basaloid epithelium with central ductal-like cells and fibroconnective stroma. KI67, SMA, CK5, CK7 specifically marked P1, P2, P3 and P4, respectively. Acinar genes were barely expressed by SBL tumor cells (**Supplementary Figure S1A, B**).

Since transcriptional programs are determined by underlying gene regulatory network (GRN), we next applied single-cell regulatory network inference and clustering (SCENIC) analysis to interrogate stable GRNs in each tumor subpopulation (24). In line with expectations, this analysis further confirmed a clear distinction in GRNs between different states (**Figure 3D**). Corresponding to its epithelium morphogenesis phenotype, activated regulons such as *PRRX1* and *MSX2* in basaloid cells were all associated with epithelial cell programming and differentiation (31, 32). As for myoepithelial-like cells, dominant regulons were transcriptional factors (TFs) that collectively control myoepithelial cell state (e.g., *MYLK, SNAI2, TP73*), crucial for maintaining mesenchymal cell state and inhibiting transition into mature luminal cells (33–35). As duct-like cells expressed ductal markers, e.g., *KRT8, KRT18*, TFs that regulated its phenotype equally promotes ductal cell differentiation and tube formation (*EHF*, *TFCP2L1*) *(* 36, 37)(**Figure 3E**). Cellular stress regulators (e.g., *MAFF, KLF9*) were also enriched in duct-like cells (**Supplementary Figure S2G**). The TFs for cycling tumor cells mainly regulate the expression of genes required for progression through the cell cycle (e.g., *BRCA1, E2F8*) (38), consistent with its proliferation phenotype.

SBL is an embryonal tumor transcriptionally similar to primitive salivary gland anlage.We next explored what developmental stage it resembles. We utilized murine embryo and postnatal salivary gland datasets comprising comprehensive cell types, including end bud, basal duct, duct, and acinar cells (22)(**Figure 3F**; **Supplementary Figures S2H, I**). Without manually and selectively refining gene signature for each cell type, we used a marker-free method of Garnett to generate an automated classifier of salivary gland (23). Classifiers by Garnett were concordant with manual classifications during self-validation (**Supplementary Figure S2J**). Using this Garnett model to train SBL tumor cells, we found most tumor cells were classified as duct or basal duct cells (**Figure 3G**). Almost no tumor cell was identified as acinar cells, in accordance with the scarce expression of acinar genes (e.g., *AQP5, NKCC1, AMY1A*) (**Figure 3C**). We further validated this finding on IHC images, which showed expression of basal and ductal genes exclusively without acinar cell differentiation. These characteristics reflect that SBL may recapitulate the pre-acinar stage of the salivary gland (**Supplementary Figures S1A, B**). Consistent with its phenotype, more myoepithelial-like tumor cells were classified as myoepithelial cells, while more duct-like tumor cells were identified as duct cells.

In order to investigate therapy-induced dynamic change of SBL tumor cells, we first compared the shift of tumor cell composition, which showed a decrease in the proportion of cycling tumor cells, demonstrating that chemotherapy significantly inhibited proliferation of tumor cells (Pearson’s chi-squared test, pvalue< 2.2e-16) (**Figure 3H**). We also examined gene expression patterns before and after chemotherapy, and identified 2,932 differentially expressed genes (DEGs) (pvalue<0.05, |logFC|>1.5) (**Figure 3I**, **Supplementary Table 6**). Functional gene ontology (GO) enrichment analysis indicated that genes with higher expression in SBL PT tumor cells were mainly enriched in cell division (*CENPF, TOP2A*), cell migration (*BST2, NR4A1, KIF2A, TJP1*) and negative regulation of cell differentiation (*YBX1, EFNB2, DNMT1*), supporting that T1 possessed a more malignant and invasive phenotype, which is consistent with gene set enrichment analysis (GSEA) (**Figures 3J, K**). In contrast, genes up-regulated in RT tumor cells were associated with negative regulation of cell population proliferation (*RARRES1, NDRG2, SPINT2*), epithelial cell differentiation (*KRT7, KRT8, KRT14, KRT15, KRT19*), muscle structure (*MYLK, TAGLN*), leukocyte mediated immunity (*GRN, CD74, SLPI*) and cell apoptosis (*HSPA1B, HSPA1A, LAMP1*) (**Figures 3J, L**), demonstrating that tumor cells turned into a less proliferated state and displaying a potentially more mature structure after treatment, consistent with the shift of tumor subpopulations.

### Uncovering Genomic and Cellular Landscape of HB

**Figure 4:**
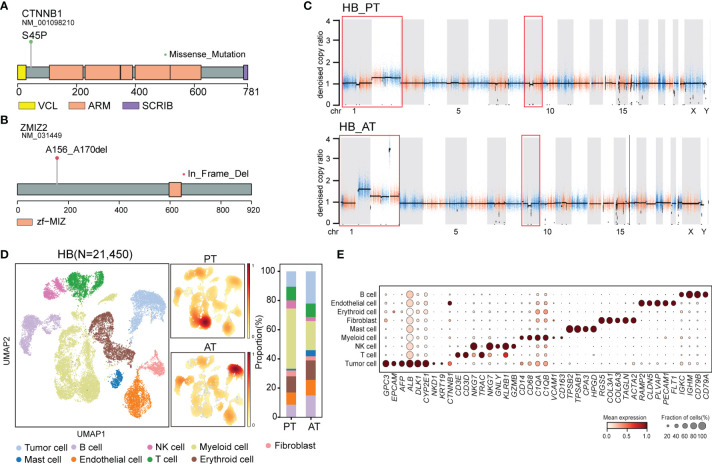
The genomic and transcriptomic landscape of HB **(A)** Lollipop plot showing *CTNNB1* p.S45P mutation. Boxes represented functional domains. VCL, interaction with VCL; ARM, Armadillo/beta-catenin-like repeats; SCRIB, interaction with SCRIB. **(B)** Lollipop plot showing *ZMIZ2* in-frame deletion. Boxes represented functional domains. Zf-MIZ, Zinc finger MIZ domain. **(C)** Scatter plots showing copy number alterations in HB PT and AT samples. **(D)** UMAP visualization of 21,450 cells from HB colored by cell types (left) and density of sample distribution(middle). Right panel showing the percentage of different cell types in HB PT and AT samples. **(E)** Dot plot for expression of cell-type-specific markers in HB. The color indicated scaled mean expression of marker genes, and the size indicated the proportion of cells expressing marker genes. HB, hepatoblastoma; PT, primary tumor; AT, tumor after treatment.

After revealing genomic and transcriptional features of SBL, we next sought to uncover characteristics of HB. For HB, WES analysis revealed that PT and AT samples both harbored 41 non-silent coding mutations, with four shared mutated genes including *CTNNB1, ANP32C*, *GOLGA8O* and *LAMB4* (**Figures 2A, B**; **Supplementary Figure S3A, B**, **Supplementary Table 3**). As the most frequent genes, *CTNNB1* p.Ser45Pro missense mutation in exon 3 affects phosphorylation sites of casein kinase I epsilon, leading to the downstream activation of Wnt pathway (**Figure 4A**) (39). Apart from *CTNNB1*, we’ve identified several cancer-related gene mutations, including *PML*, *HSP90AA1*, *BAZ1A*, *ARHGAP5*, *LHFPL6* in the primary tumor of HB, which may also contribute to the carcinogenesis of HB (40). Mutated genes in HB were enriched in response to DNA damage, epithelium development, Wnt and PI3K-Akt signaling pathways (**Supplementary Figures S3C, D**).

Though no common somatic variants were found between primary tumors of SBL and HB, in-frame deletion of *PAN3* and *ZMIZ2* were identified in both tumor samples after treatment (**Figures 2A, B**). *PAN3*, a regulatory subunit of the PAN2-PAN3 complex, was involved in the metabolism of messenger RNA (41). As one of PIAS-like proteins, ZMIZ2 has been revealed to interact with β-catenin physically, augmenting β-catenin-mediated transcription and tumor cell growth (42, 43). The disruption of ZMIZ2 in tumor tissues of both SBL and HB may lead to down-regulation of β-catenin downstream targets and inhibition of the Wnt signaling pathway after treatment (**Figure 4B**).

In terms of somatic copy number alterations, whole chromosome 2 gain and segmental 9p12-q21 loss were shared in both PT and AT samples of HB. Chromosome 1p12-1q21 deletion was exclusively detected in the PT sample while 1p gain was identified in AT sample of HB (**Figure 4C**; **Supplementary Table 4**), suggesting tumor evolution after chemotherapy.

Following revealing its genomic features, we next focused on the transcriptional characteristics of HB. After quality control, we obtained 21,450 single-cell transcriptomes from PT and AT samples of HB. Based on the canonical marker gene expression, 3,085 cells were annotated as tumor cells (*GPC3, EPCAM, AFP, ALB, DLK1*), while 18,365 cells were designed as non-tumor cells (**Figures 4D, E**) (44, 45). We further confirmed the identity of tumor cells by evaluation of inferred segmental chromosomal alterations. Consistent with WES findings, PT tumor cells showed chromosome 1q loss and 2 gain, while AT tumor cells exhibited chromosome 1q and 2 gain (**Supplementary Figure S3E**).

**Figure 5:**
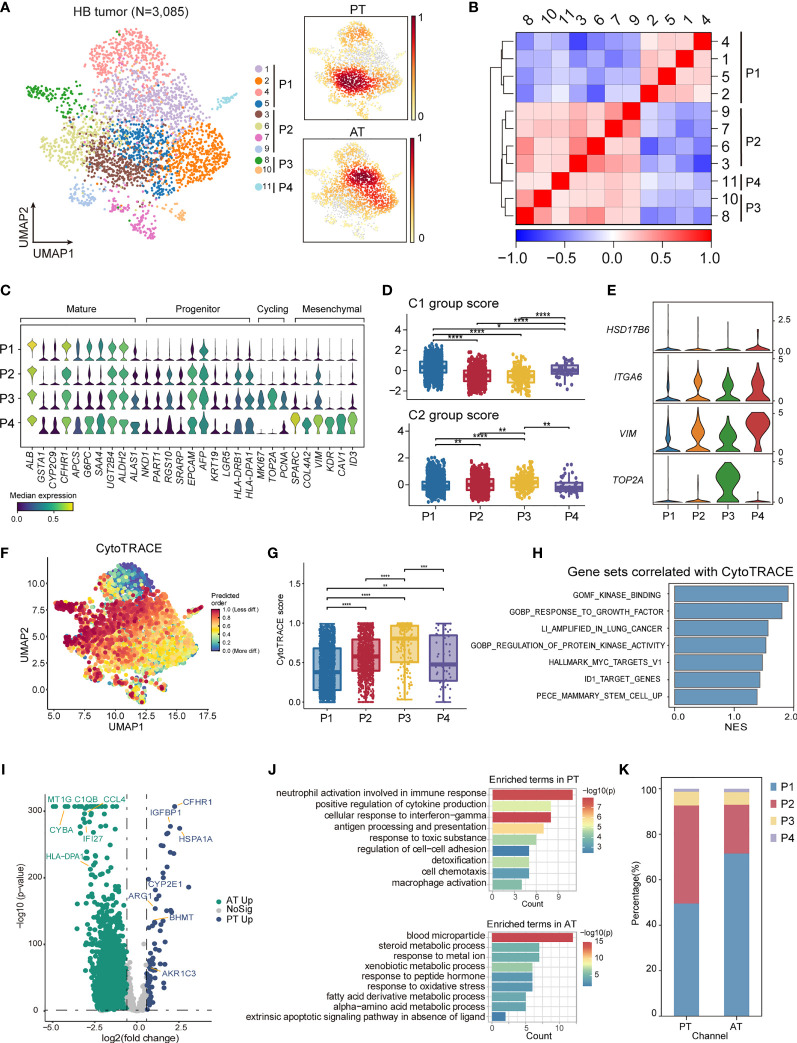
The intratumoral heterogeneity within HB malignant cells. **(A)** UMAP visualization of 3,085 tumor cells from HB colored by clusters (left) and density of sample distribution(right). **(B)** Heatmap displaying the Pearson correlation coefficients calculated between average gene expressions of tumor clusters. **(C)** Violin plots showing the expression distribution of selected genes involved in cell cycle genes, and markers specific to mature hepatocytes, hepatoblasts, and mesenchymal cells in HB tumor subtypes. **(D)** Boxplots showing the expression score of HB C1 and C2 group gene sets curated from Cairo et al. in HB tumor subtypes. *P < 0.05, **P < 0.01, ***P < 0.001, ****P < 0.0001, two-sided Wilcoxon test. **(E)** Violin plots showing the expression of four-gene signature curated from Hooks et al. in HB tumor subtypes. **(F)** UMAP visualization of CytoTRACE values of HB malignant cells. **(G)** Boxplots showing CytoTRACE values in HB tumor subtypes. *P < 0.05, **P < 0.01, ***P < 0.001, ****P < 0.0001, two-sided Wilcoxon test. **(H)** GSEA analysis of genes correlated with high CytoTRACE values (associated with immaturity). **(I)** Volcano plot showing differentially expressed genes between HB PT (green dots) and RT tumor cells (blue dots). The names of selected important genes are indicated in the plots. **(J)** Bar chart showing the enrichment of GO terms, based on top50 upregulated genes in PT or AT malignant cells. **(K)** Bar plot indicating the percentage of tumor subtypes in HB PT and AT samples. HB, hepatoblastoma; PT, primary tumor; AT, tumor after treatment.

To elucidate various expression states of malignant cells within HB, we performed unsupervised clustering of all tumor cells (**Figure 5A**). A total of 10 subclusters were generated, which can be aggregated into 4 expression programs using hierarchical clustering based on expression profiles (**Figures 5B, C**; **Supplementary Table 7**). The 16-gene signature developed by Cairo et al. and the 4-gene signature developed by Hooks et al., which have been used to discriminate mature and invasive HB, was also evaluated for each program (8, 9) (**Figures 5D, E**).

P1, which constituted a major fraction of HB tumor cells and had the highest C1 group score, exhibited a more mature hepatocyte-like phenotype, expressing the highest level of *ALB* as well as liver metabolism genes (e.g., *CYP2E1*, *GSTA1*, *G6PC*) (**Figures 5C, D**; **Supplementary Figure S3F**) (46). Therefore, P1 corresponded to liver features at later stages of fetal development and the fetal histology pattern of HB. Unlike P1, P2 expressed higher expression of liver progenitor markers *AFP, EPCAM, KRT19*, *LGR5*, and abundant immune-related genes, including MHC class II antigen presentation genes (e.g., *HLA-DRA, HLA-DRB1*). P3 reflected distinguished cycling cells, enriching cell-cycle genes (e.g., *MKI67, TOP2A, HMGB2*). P2 and P3 had higher C2 group scores and relatively lower C1 group scores. And P3 may resemble the C2a group with enhanced expression of *TOP2A* (**Figures 5C–E**; **Supplementary Figure S3F**). The features suggested that P2 and P3 obtained more invasive signatures, recapitulating earlier stages of liver development and embryonal histology pattern of HB. Immunohistology analysis also confirmed the existence of P1 and P2/P3 programs, reflecting the co-occurrence of fetal and embryonal patterns (**Supplementary Figure S1C**).

In contrast with the other three programs, a fourth program, P4, which was only comprised of a minor fraction of malignant cells, presented moderate expression of both C1 and C2 group genes. Nevertheless, P4 up-regulated mesenchymal features (e.g., *SPARC*, *COL4A2*) as well as C2b signature gene *VIM*, hence annotated as a group of mesenchymal-like cells (**Figures 5C–E**; **Supplementary Figure S3F**). In the meantime, enhanced expression of early hepatoblast markers (e.g., *KDR*, *CAV1*, *ID3*) was also found in P4, reminiscent of a subpopulation of ID3+ hepatoblasts restricted to the development stages of liver bud (47–49).

We validated the classification of different transcriptional programs by SCENIC regulon analysis. We selected the regulons with the highest regulon specificity score for each program (**Supplementary Figures S3G, H**). Our analysis identified *HNF4A* and *NR1I3* as specific regulons associated with P1, both of which were well-characterized regulators for core hepatic genes (50, 51). For P2, the most specific regulons included *HMGA2* and *MYCN*. The deregulated MYCN-HMGA2-LIN28B pathway, which contributed to tumor development and progression (52, 53), was activated in P2, further strengthening its invasive phenotype. P3 was associated with cell-cycle regulators, e.g., *FOXM1*, *BRCA1*. Consistent with its mesenchymal state, P4 was enriched for tumor epithelial-mesenchymal transition regulons, e.g., *SOX18, ZEB1* and *NFIB* (54–56).

To further define divergent differential status in HB, we used CytoTRACE (57) to predict the developmental potential for each tumor program. In accordance with immature transcriptional phenotype, cells in P2 and P3 are with the highest CytoTRACE score (**Figures 5F, G**). Gene sets positively correlated with HB immaturity included MYC targets, kinase activity, and stem cells (**Figure 5H**).

Finally, to gain insights into the transcriptional changes of HB tumor cells after chemotherapy, we performed a DEG analysis between PT and AT malignant cells (**Figure 5I**; **Supplementary Table 8**). We observed an enrichment of genes involved in cell adhesion and immune-response pathways (e.g., antigen processing and presentation, cell chemotaxis) in PT which were signatures of P2, whereas the genes up-regulated in AT mainly belonged to normal liver metabolism pathways (e.g., blood microparticle, steroid metabolic process), the signatures for P1 (**Figure 5J**). This led to the hypothesis that the composition of tumor cells could change after treatment. We next compared the proportion of each tumor group in HB PT and AT. Consistent with DEG analysis, the proportion of P1 was higher in AT than in PT, while the proportion of P2 was higher in PT. These results suggested a more mature phenotype was induced by chemotherapy. (**Figure 5K**). H&E further confirmed this finding, exhibiting mixed fetal and embryonal patterns in PT were transformed into fetal-dominant histology patterns in AT (**Figure 1C**; **Supplementary Figures S1C, D**).

### Investigation of Similarities between Synchronous SBL and HB

**Figure 6:**
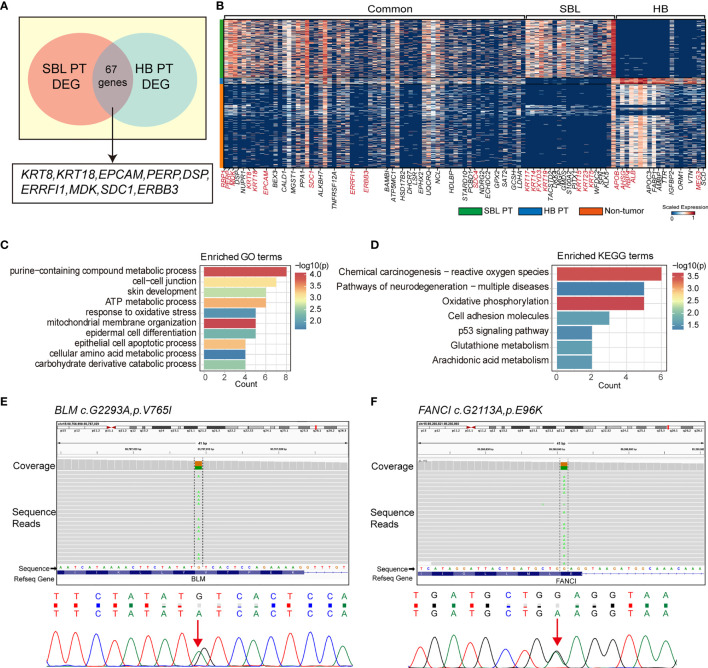
Transcriptional similarities between SBL and HB malignant cells. **(A)** Venn diagram displaying shared signature genes between SBL and HB malignant cells. **(B)** Heatmap showing the expression of shared genes and selected top20 unique signature genes in SBL and HB malignant cells. **(C)** Bar chart showing the enrichment of GO terms, based on shared signature genes between SBL and HB malignant cells. **(D)** Bar chart showing the enrichment of KEGG terms, based on shared signature genes between SBL and HB malignant cells. **(E)** IGV screenshot (upper) and DNA sequence chromatogram of Sanger sequencing (bottom) demonstrating *BLM* c.G2293A, pV765I germline mutation. **(F)** IGV screenshot (upper) and DNA sequence chromatogram of Sanger sequencing (bottom) demonstrating *FANCI* c.G2113A, pE96K germline mutation. SBL, sialoblastoma; HB, hepatoblastoma; PT, primary tumor; DEG, differentially expressed gene.

The salivary gland and liver have different embryonic origins. It’s been suggested salivary gland epithelium arises from the oral ectoderm, whereas liver parenchyma comes from the foregut endoderm (58, 59). Despite different origins, they share functional similarities as accessory organs of the digestive system, playing a major function in digestion. Recent studies suggested that adult salivary gland progenitors can be induced into functional hepatocytes and pancreatic cells both *in vitro* and *in vivo* culture conditions (60, 61). These prompted us to investigate the similarities of SBL and HB. By calculating the DEGs of each PT versus non-tumor cells (stromal and immune cells) respectively, we searched for overlapping genes between the signature genes of SBL and HB. This analysis found 67 DEGs were shared between SBL and HB malignant cells (**Figures 6A, B**). As one of the hallmark traits of the tumor, deregulating cellular metabolism (ATP metabolic, amino acid, and carbohydrate metabolic process) pathways were up-regulated (**Figures 6C, D**). Apart from these, SBL and HB both showed enhanced expression of epithelial markers (e.g., *KRT8, KRT18, PERP, DSP*) and essential embryo development genes (e.g., *SDC1, MDK, ERBB3*) *(* 62–64), demonstrating that disruption of normal embryo epithelial development gave rise to the tumorigenesis of both tumors. In spite of transcriptional similarities, there still existed apparent differences between SBL and HB. SBL uniquely expressed cytokeratins specific to salivary duct and basal cells (e.g., *KRT5, KRT15, KRT23, KRT17, KRT19*) while HB showed significant expression of liver metabolism genes (e.g., *APOE, APOC3, ALB, AHSG*), reflecting signatures of original organs (**Figure 6B**; **Supplementary Table 9**).

Out of its rarity of synchronous multiple primary tumors in a single patient, especially for children, we proposed that this patient may carry pathogenic germline mutations that predispose him to higher cancer risk. Based on known cancer predisposing genes curated from COSMIC database (40) and other pan-cancer germline genomic studies (65–67), we found a total of 6 damaging or potentially damaging heterozygous germline variants including missense mutation of *BLM* and *FANCI* (**Table 2**). Sanger sequencing was performed for variant validation (**Figures 6E, F**; **Supplementary Figure S4A–D**). *BLM* and *FANCI* play an essential role in homologous recombination (HR)-mediated repair of DNA interstrand cross-links, the mutation of which resulted in the deficiency of DNA repair and lead to genome instability (68). The biallelic germline mutations of *BLM* and *FANCI* resulted in Bloom syndrome and Fanconi anemia (FA), respectively, characterized by congenital disabilities and cancer predisposition (69, 70). Recently, several studies have also revealed the gene-dose effect (haploinsufficiency) of *BLM* and *FA* family genes may be associated with cancer predisposition (71–73). *BLM* haploinsufficiency led to an early onset of murine T cell lymphoma in response to challenge with leukemia virus (72). Carriers of deleterious *BLM* mutations are at increased risk to develop colorectal cancer and breast cancer (71, 73). Heterozygous mutations in FA-family genes are associated with hereditary breast cancer and familial ovarian cancer (73, 74). Since BLM helicase and FA proteins work concurrently in the DNA repair and maintenance of genome stability, we suppose the patient with predicted deleterious heterozygous germline mutations of two genes may be sensitive to DNA-damaging agents and thus have an increased cancer risk. In addition, we sought to identify potential pathogenic germline CNVs (**Supplementary Table 10**). Two CNVs (chromosome 8q24.22-q24.3 and 11p11.2-p12 deletion) encompassed known germline cancer-predisposing genes (*RECQL4* and *EXT2*, respectively) (65–67) and were not appeared in the DGV database, indicating both CNVs were probably damaging (**Supplementary Figure S4E, F**). However, since the infant was an abandoned child, we could not collect his family history. More cases are needed to validate these findings.

**Table 2:** Potential pathogenic germline variants.

Gene Symbol	Position	Mutation	Allele Frequency(gnomAD)	SIFT	PolyPhen	CADDPHRED	CLNDN
***BLM***	chr15:90767009-90767009	c.G2293A; p.Val765Ile	0.0003	deleterious	Possibly damaging	25.8	Hereditary_breast_and_ovarian_cancer_syndrome|Bloom_syndrome|Hereditary_cancer-predisposing_syndrome|not_provided
***GOLGA5***	chr14:92810322-92810322	c.T1061C; p.Leu354Pro	0.0004	deleterious	Probably damaging	28.9	.
***USP44***	chr12:95524686-95524686	c.G1727A; p.Arg576Gln	0.000012	deleterious	Probably damaging	31	.
***USH2A***	chr1:215965369-215965369	c.T7068G; p.Asn2356Lys	0.0008	deleterious	Possibly damaging	23	Usher_syndrome,_type_2A|Retinitis_pigmentosa_39|not_specified|not_provided
***EPS15***	chr1:51394387-51394387	c.G2113A; p.Asp705Asn	0.0014	deleterious	Probably damaging	25.2	.
***FANCI***	chr15:89260841-89260841	c.G286A; p.Glu96Lys	0.0017	deleterious	Probably damaging	33	Fanconi_anemia,_complementation_group_I|Fanconi_anemia

## Discussion

In the current study, we presented a rare case of synchronous SBL and HB, and constructed a cellular and molecular landscape of both tumors from a single-cell level, providing a valuable resource for synchronous tumor research.

In the first part, we focused on the genome and transcriptomic features of SBL. SBL is a rare embryonal salivary gland tumor, first reported by Vawter and Tefft in 1966 (75). Only around 80 cases of SBL have been reported around the world, with few studies elucidating its molecular characteristics (76). We identified a hotspot mutation *PIK3CA* E542K and chromosome 5q34-5q35.3 loss in PT, which might play an important role in tumorigenesis. Previous studies of SBL have identified 47, XX, del(3)(q13), inv(9) (p11q12)c, +?r[5]/46,XX,inv(9) (p11q12)c[41] in a female patient with submandibular SBL (77), and FGFR2 p.Cys382Arg activated somatic mutation in a child with unresectable SBL (78). Our study did not detect recurrent patterns, suggesting potential genetic heterogeneity of this disease. Nevertheless, a gain of function on the FGFR2 protein results in activation of downstream pathways, including PI3K/AKT, strengthening the importance of PI3K signaling pathway in SBL (79). Through scRNA-seq, we uncovered intra-tumoral heterogeneity of SBL. Tumor cells can be divided into 4 different subtypes, including basaloid cells (*KRT5, KRT14*), myoepithelial cells (*ACTA2, MYL*), cycling cells (*MKI67, TOP2A*) and duct-like cells (*KRT7, KRT19*). After chemotherapy, tumor cells were induced into a more mature phenotype, indicating the effectiveness of the current regimen. By comparing SBL tumor cells with its normal counterparts, we found SBL may resemble a pre-acinar stage of the salivary gland, while few cells are classified as acinar cells. Immunostaining results also confirmed this finding.

In the second part, we turned to explore the characteristics of HB. Canonical *CTNNB1* p.Ser45Pro missense mutation, as well as several other cancer-related genes (e.g., *PML*, *HSP90AA1*, *BAZ1A*, *ARHGAP5*, *LHFPL6*), were detected in the PT sample. No common somatic variant was found between SBL and HB PT samples. Previous transcriptomic analysis identified 3 major subtypes of HB samples, including C1, C2a, and C2b, revealing inter-tumoral heterogeneity(9). Our results supported this notion and expanded it to intra-tumoral heterogeneity. Our analysis indicated four subtypes of HB tumor cells, with P1, P3, P4 corresponding to C1, C2a, and C2b, respectively. P1 represented fetal-hepatocyte-like tumor cells in this tumor sample, while P2/P3 represented embryonal-hepatocyte-like tumor cells.

Finally, we investigated the similarities of both tumors and detected hazardous germline mutations of this patient. SBL and HB both showed enhanced expression of epithelial markers (e.g., *KRT8, KRT18* and embryo development genes (e.g., *SDC1, MDK*), suggesting the interruption of normal epithelial development. In addition, we found 6 damaging or potentially damaging heterozygous germline variants. Deleterious germline mutation *BLM* and *FANCI* play an essential role in DNA repair. Heterozygous mutation of both genes may predispose the patient to higher cancer risk, contributing to the tumorigenesis of SBL and HB. However, despite several studies suggesting that heterozygous mutations in DNA repair genes may predispose carriers to increased cancer susceptibility (71–73), this concept still remains controversial and association between genes and specific cancer type warranted further validation. Though predicted to be deleterious and pathogenic by in-silico algorithms, the germline variants of *BLM* and *FANCI* identified in this study were missense mutations with unclear impact on protein function, which requires further validation. In addition, we identified potentially damaging chr8q24.22-q24.3 and chr11p11.2-p12 deletion affecting tumor suppressor gene *RECQL4* and *EXT2*, respectively (80, 81). In 1999, Choi HJ et al. reported a case of chr1q deletion with synchronous SBL and HB. We’ve also detected germline chr1q12-q21.2 deletion (**Supplementary Table 10**) and somatic chr1p12-1q21 deletion in the primary HB tumor tissue, indicating that chr1q deletion may participate in the pathogenesis of this disease. Rigorous functional experiments and more cases are needed to verify our findings.

As tumors from different organs, the synchronous occurrence of SBL and HB has rarely been revealed, with only 5 previously reported cases (**Table 3**) (12–16). After summarizing all cases, we found that almost all patients were prenatally diagnosed with well-differentiated histology, further revealing the embryonal origin of both tumors and providing clues into the development similarities of salivary gland and liver. The salivary gland and liver are accessory organs of the digestive system, sharing functional similarities in food digestion. The development of both organs during embryo development consisted of primitive bud formation followed by branching morphogenesis. Interaction of epithelium and mesenchyme plays a vital role in the maturation of both organs. More interestingly, epithelial cells from the salivary gland can be induced into functional hepatocytes *in vitro*, which can alleviate severe acute liver damage caused by carbon tetrachloride in SCID mouse (82). Several studies have proposed that both organs might share the same embryonal origin. However, recent genetic tracing results indicated the ectodermal origin of the salivary gland and the endodermal origin of the liver (58, 59). More studies are needed to explain the potential developmental similarities of both organs. Though with limited cases, there might be a slight male preponderance for synchronous SBL and HB, with four male patients and only one female patient. This could be partially explained by male predisposition in both SBL (Male : Female=~1.3:1) (1, 4, 83, 84) and HB (Male : Female=1.4-2:1) (85–87). In addition, two reported cases adopted neoadjuvant chemotherapy (cisplatin, vincristine, 5-fluorouracil or vincristine, actinomycin, cyclophosphamide) before surgical resection. Though the volume of HB decreased, SBL showed no response to treatment with a static size. In our case, we utilized an intermediate neuroblastoma regimen (carboplatin, cyclophosphamide/etoposide, and doxorubicin), which were effective in decreasing tumor volume and inducing a more mature phenotype for both tumors. This therapy may provide a reference for treating synchronous SBL and HB.

**Table 3:** Summary of synchronous pediatric sialoblastoma and hepatoblastoma cases.

No.	1	*2*	*3*	*4*	*5*	*6*
***Year***	1999	2000	2009	2012	2021	2022
***Country***	Korea	USA	South Africa	China	Argentina	China
***Sex***	N/A	Female	Male	Male	Male	Male
***Age at Dx***	N/A	37W GA	34W GA	28W GA	20W GA	6 months
***Tumor size*** ***(cm^3^)***	N/A	SBL: 11×8.5×8.5; HB: 5.3×2.2×3.2;	SBL: 8×4×3;	SBL: ~8; HB: ~5;	SBL: ~11.4×0.74×8; HB: ~ 4×2.8;	SBL:6.6×6.1×4.3; HB:9.1×8.5×8.5;
***Primary Site***	N/A	SBL: right parotid gland; HB: left lobe, and nodules in the porta hepatis and right lobe	SBL: right parotid gland; HB: nodules in the left and right lobes;	SBL: left submandibular gland; HB: right lobe;	SBL: right parotid gland; HB: left lobe;	SBL: left parotid gland; HB: left and right lobes
***Lab tests*** ***(ng/ml)***	N/A	AFP 432,240↑	N/A	AFP 671,500↑	AFP 365,121↑	AFP 121,000↑ CEA 5.79↑ NES 30.30↑
***Histopathological results***	N/A	SBL; HB: fetal-type	SBL; HB: fetal-type	SBL and HB	SBL; HB: mixed embryonal/fetal subtype	SBL; HB: mixed embryonal/fetal subtype
***Germline mutation/*** ***CNV***	chr1q del	N/A	N/A	No deletion or duplication syndromes	N/A	*BLM* and *FANCI* mutation; chr1q12-21.2, chr8q24.22-q24.3, chr11p11.2-p12 del
***Treatment***	N/A	SBL: tumor resection; HB: tumor resection and chemotherapy (cisplatin, vincristine, 5-fluorouracil);	N/A	Chemotherapy (cisplatin, vincristine, and 5-fluorouracil) followed by tumor resection	Neoadjuvant chemotherapy (vincristine, actinomycin, cyclophosphamide) followed by tumor resection and chemotherapy (cisplatin)	Neoadjuvant chemotherapy followed by tumor resection and chemotherapy (Carboplatin, cyclophosphamide/etoposide, doxorubicin)
***Chemotherapy Response***	N/A	No recurrence or metastasis	N/A	SBL: static in size; HB: reduced in size;	Poor response	Reduced in size
***Follow-up***	N/A	5.5 years	N/A	8 months	2 years	32 months
***Outcome***	N/A	Alive	Dead (septicemia)	Alive	Alive	Alive
***Ref***	(12)	(13)	(14)	(15)	(16)	This study

↑, elevated level of tumor markers; N/A, not available.

Our study also has its limitations. First, out of the rarity of synchronous SBL and HB, our analysis was confined to only one case and did not have enough sample size. Results may not be comprehensive and offer abundant information. Much remains to be gained by enlarging the cohort. Second, because of the specificity of the case, we were not able to obtain a family history of the patient and further investigate the inheritance pattern of germline mutations. Also due to limitations of WES technology (e.g., non-uniform read-depth distribution and restricted to exonic regions), it’s difficult to accurately detect large CNVs or structural variants (SVs) in genomic areas. Therefore, identified potential deleterious CNVs could be confirmed with orthogonal methods. The possible role of pathogenic SVs should be further explored if whole genome sequencing data was available. Future efforts in collecting more cases and entire family history will facilitate the identification of genetic abnormalities of the disease.

## Data Availability Statement

The datasets presented in this study can be found in online repositories. The names of the repository/repositories and accession number(s) can be found below: Gene Expression Omnibus (GEO), accession number GSE166345, and Genome Sequence Archive (GSA) for Human in National Genomics Data Center (NGDC), accession number HRA000610.

## Ethics Statement

The studies involving human participants were reviewed and approved by Children’s Hospital of Fudan University. Written informed consent to participate in this study was provided by the participants’ legal guardian/next of kin.

## Author Contributions

RY, YZ, and YL contributed to conception and design, data acquisition, analysis and interpretation, drafted and critically revised the manuscript. C-JY, L-DM, and D-QC contributed to data curation and investigation. S-WH and S-YD contributed to materials collection and literature review. C-BD, LC, GC, K-RD, and SZ contributed to manuscript review and editing. JL, WY, and RD contributed to the experiment design and critically revised the manuscript. All authors contributed to the article and approved the submitted version.

## Funding

This work was supported by the Cyrus Tang Foundation (No. ZSBK0070), Shanghai Municipal Key Clinical Specialty (No. shslczdzk05703), National Natural Science Foundation of China (No. 82072782) and Shanghai Municipal Health Commission (No. EK112520180301).

## Conflict of Interest

The authors declare that the research was conducted in the absence of any commercial or financial relationships that could be construed as a potential conflict of interest.

## Publisher’s Note

All claims expressed in this article are solely those of the authors and do not necessarily represent those of their affiliated organizations, or those of the publisher, the editors and the reviewers. Any product that may be evaluated in this article, or claim that may be made by its manufacturer, is not guaranteed or endorsed by the publisher.
