# Supplementary material for: The Cellular and Molecular Landscape of Synchronous Pediatric Sialoblastoma and Hepatoblastoma
Source: Front Oncol. 2022 Jul 4;12:893206. doi: 10.3389/fonc.2022.893206 (PMC9289541; doi:10.3389/fonc.2022.893206)
Supplement: Supplementary file 1 [file DataSheet_1.pdf]

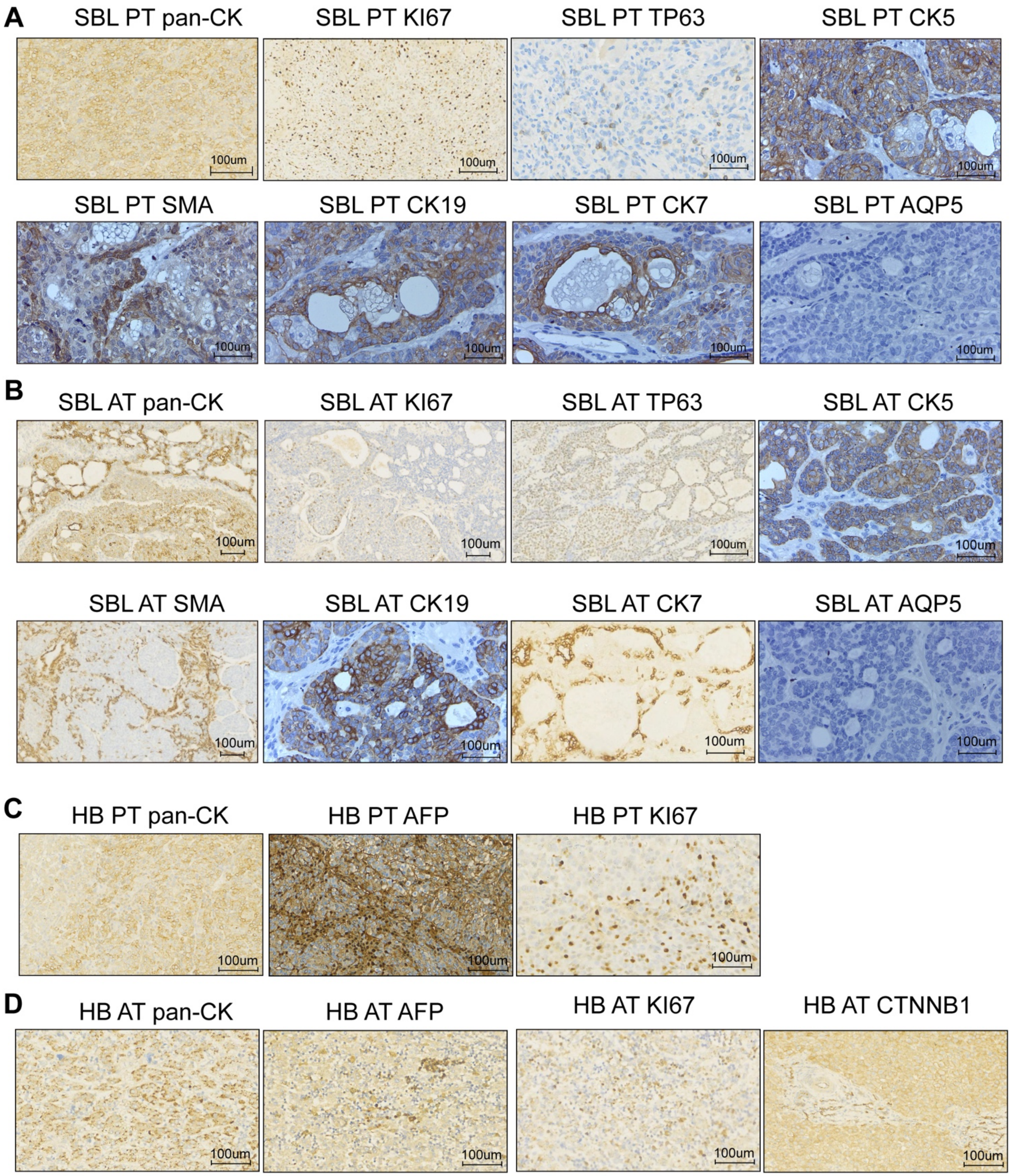

Supplementary Figure S1 IHC images of SBL and HB tumor samples. (A-B) IHC images of SBL PT and AT samples stained for the epithelial marker (pan-CK), cycling marker (KI67), basal duct markers (TP63, CK5), myoepithelial-markers (a-SMA), duct marker (CK19, CK7), and acinar marker (AQP5). Scale bar, 100 uM. **(C-D)** IHC images of HB PT and AT samples stained for the epithelial marker (pan-CK), cycling marker (KI67), liver progenitor marker (AFP), and CTNNB1. Scale bar, 100 uM.

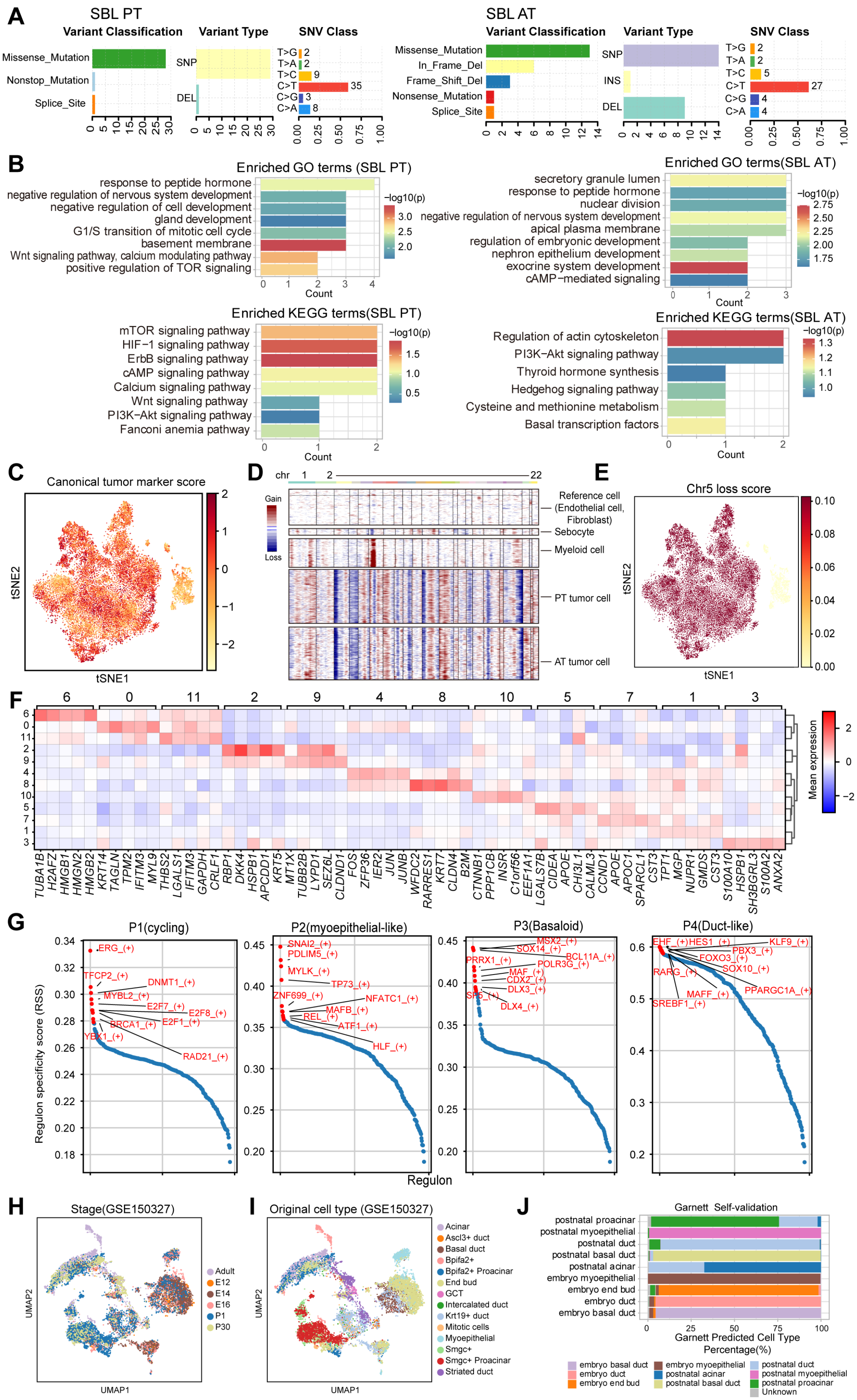

A
SBL PT
Variant Classification
Missense_Mutation
Nonstop_Mutation
Splice_Site
Variant Type
SNP
DEL
SNV Class
T>G 2
T>A 2
T>C 9
C>T 35
C>G 3
C>A 8
SBL AT
Variant Classification
Missense_Mutation
In_Frame_Del
Frame_Shift_Del
Nonsense_Mutation
Splice_Site
Variant Type
SNP
INS
DEL
SNV Class
T>G 2
T>A 2
T>C 5
C>T 27
C>G 4
C>A 4
B
Enriched GO terms (SBL PT)
response to peptide hormone
negative regulation of nervous system development
negative regulation of cell development
gland development
G1/S transition of mitotic cell cycle
basement membrane
Wnt signaling pathway, calcium modulating pathway
positive regulation of TOR signaling
Count
−log10(p)
Enriched GO terms(SBL AT)
secretory granule lumen
response to peptide hormone
nuclear division
negative regulation of nervous system development
apical plasma membrane
regulation of embryonic development
nephron epithelium development
exocrine system development
cAMP−mediated signaling
Count
−log10(p)
Enriched KEGG terms(SBL PT)
mTOR signaling pathway
HIF−1 signaling pathway
ErbB signaling pathway
cAMP signaling pathway
Calcium signaling pathway
Wnt signaling pathway
PI3K−Akt signaling pathway
Fanconi anemia pathway
Count
−log10(p)
Enriched KEGG terms(SBL AT)
Regulation of actin cytoskeleton
PI3K−Akt signaling pathway
Thyroid hormone synthesis
Hedgehog signaling pathway
Cysteine and methionine metabolism
Basal transcription factors
Count
−log10(p)
C
Canonical tumor marker score
tSNE1
tSNE2
D
chr 1 2 22
Gain
Loss
Reference cell (Endothelial cell, Fibroblast)
Sebocyte
Myeloid cell
PT tumor cell
AT tumor cell
E
Chr5 loss score
tSNE1
tSNE2
F
Mean expression
TUBA1B H2AFZ HMGB1 HMGN2 HMGB2 KRT14 TAGLN TPM2 IFITM3 MYL9 THBS2 LGALS1 IFITM3 GAPDH CRLF1 RBP1 DKK4 HSPB1 APCDD1 KRT5 MT1X TUBB2B LYPD1 SEZ6L CLDND1 FOS ZFP36 IER2 JUN JUNB WFDC2 RARRES1 KRT7 CLDN4 B2M CTNNB1 PPP1CB INSR C1orf56 EEF1A1 LGALS7B CIDEA APOE CHI3L1 CALML3 CCND1 APOE APOC1 SPARCL1 CST3 TPT1 MGP NUPR1 GMDS CST3 S100A10 HSPB1 SH3BGRL3 S100A2 ANXA2
G
P1(cycling)
P2(myoepithelial-like)
P3(Basaloid)
P4(Duct-like)
Regulon specificity score (RSS)
Regulon
ERG_(+)
TFCP2_(+)
DNMT1_(+)
MYBL2_(+)
E2F7_(+)
E2F8_(+)
E2F1_(+)
BRCA1_(+)
YBX1_(+)
RAD21_(+)
SNAI2_(+)
PDLIM5_(+)
MYLK_(+)
TP73_(+)
ZNF699_(+)
NFATC1_(+)
MAFB_(+)
REL_(+)
ATF1_(+)
HLF_(+)
MSX2_(+)
SOX14_(+)
BCL11A_(+)
PRRX1_(+)
POLR3G_(+)
MAF_(+)
CDX2_(+)
DLX3_(+)
SP6_(+)
DLX4_(+)
EHF_(+)
HES1_(+)
KLF9_(+)
PBX3_(+)
FOXO3_(+)
SOX10_(+)
RARG_(+)
PPARGC1A_(+)
MAFF_(+)
SREBF1_(+)
H
Stage(GSE150327)
Adult
E12
E14
E16
P1
P30
UMAP1
UMAP2
I
Original cell type (GSE150327)
Acinar
Ascl3+ duct
Basal duct
Bpifa2+
Bpifa2+ Proacinar
End bud
GCT
Intercalated duct
Krt19+ duct
Mitotic cells
Myoepithelial
Smgc+
Smgc+ Proacinar
Striated duct
UMAP1
UMAP2
J
Garnett Self-validation
postnatal proacinar
postnatal myoepithelial
postnatal duct
postnatal basal duct
postnatal acinar
embryo myoepithelial
embryo end bud
embryo duct
embryo basal duct
Garnett Predicted Cell Type
Percentage(%)
embryo basal duct
embryo duct
embryo end bud
embryo myoepithelial
postnatal acinar
postnatal basal duct
postnatal duct
postnatal myoepithelial
postnatal proacinar
Unknown

Supplementary Figure S2 Genomic and transcriptomic features of SBL(A) Bar plots showing mutated genes in SBL PT and AT samples categorized by variant classification, variant type, and SNV classes. **(B)** Bar chart showing the enrichment of GO and KEGG terms, based on mutated genes in SBL PT and AT samples.**(C)** UMAP visualization of SBL 22,107 cells colored by expression score of tumor marker genes.**(D)** Heatmap showing the inferred CNV profiles in malignant cells and their comparison with non-malignant cells.**(E)** UMAP visualization of SBL 22,107 cells colored by chromosome 5 loss score.**(F)** Heatmap showing the expression of top 5 signature genes for each tumor cluster shown in Figure 3A.**(G)** Rank for regulons in each SBL tumor subtype based on regulon specificity score (RSS). **(H-I)** UMAP visualization of murine salivary gland datasets (GSE150327) colored by original stages and cell types, respectively. **(J)** Bar chart comparing the original labels with cell-type assignments by Garnett. The different colors indicated different cell types.

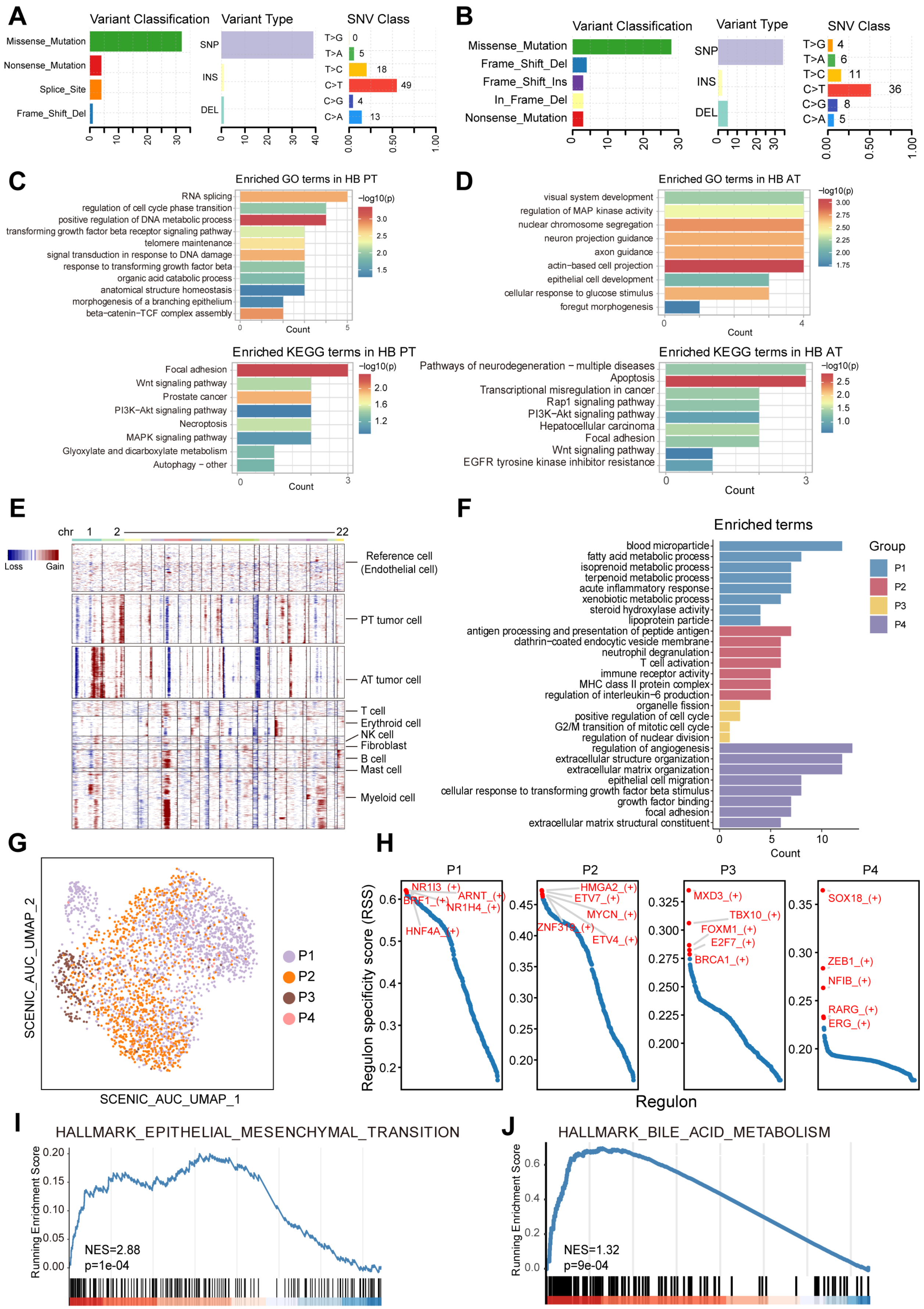
A
Variant Classification
Variant Type
SNV Class
Missense_Mutation
Nonsense_Mutation
Splice_Site
Frame_Shift_Del
SNP
INS
DEL
T>G 0
T>A 5
T>C 18
C>T 49
C>G 4
C>A 13
B
Variant Classification
Variant Type
SNV Class
Missense_Mutation
Frame_Shift_Del
Frame_Shift_Ins
In_Frame_Del
Nonsense_Mutation
SNP
INS
DEL
T>G 4
T>A 6
T>C 11
C>T 36
C>G 8
C>A 5
C
Enriched GO terms in HB PT
RNA splicing
regulation of cell cycle phase transition
positive regulation of DNA metabolic process
transforming growth factor beta receptor signaling pathway
telomere maintenance
signal transduction in response to DNA damage
response to transforming growth factor beta
organic acid catabolic process
anatomical structure homeostasis
morphogenesis of a branching epithelium
beta−catenin−TCF complex assembly
Count
−log10(p)
D
Enriched GO terms in HB AT
visual system development
regulation of MAP kinase activity
nuclear chromosome segregation
neuron projection guidance
axon guidance
actin−based cell projection
epithelial cell development
cellular response to glucose stimulus
foregut morphogenesis
Count
−log10(p)
Enriched KEGG terms in HB PT
Focal adhesion
Wnt signaling pathway
Prostate cancer
PI3K−Akt signaling pathway
Necroptosis
MAPK signaling pathway
Glyoxylate and dicarboxylate metabolism
Autophagy − other
Count
−log10(p)
Enriched KEGG terms in HB AT
Pathways of neurodegeneration − multiple diseases
Apoptosis
Transcriptional misregulation in cancer
Rap1 signaling pathway
PI3K−Akt signaling pathway
Hepatocellular carcinoma
Focal adhesion
Wnt signaling pathway
EGFR tyrosine kinase inhibitor resistance
Count
−log10(p)
E
chr 1 2 22
Loss Gain
Reference cell (Endothelial cell)
PT tumor cell
AT tumor cell
T cell
Erythroid cell
NK cell
Fibroblast
B cell
Mast cell
Myeloid cell
F
Enriched terms
Group
P1
P2
P3
P4
blood microparticle
fatty acid metabolic process
isoprenoid metabolic process
terpenoid metabolic process
acute inflammatory response
xenobiotic metabolic process
steroid hydroxylase activity
lipoprotein particle
antigen processing and presentation of peptide antigen
clathrin−coated endocytic vesicle membrane
neutrophil degranulation
T cell activation
immune receptor activity
MHC class II protein complex
regulation of interleukin−6 production
organelle fission
positive regulation of cell cycle
G2/M transition of mitotic cell cycle
regulation of nuclear division
regulation of angiogenesis
extracellular structure organization
extracellular matrix organization
epithelial cell migration
cellular response to transforming growth factor beta stimulus
growth factor binding
focal adhesion
extracellular matrix structural constituent
Count
G
SCENIC_AUC_UMAP_2
SCENIC_AUC_UMAP_1
P1
P2
P3
P4
H
P1
P2
P3
P4
Regulon specificity score (RSS)
NR1I3_(+)
ARNT_(+)
BRF1_(+)
NR1H4_(+)
HNF4A_(+)
HMGA2_(+)
ETV7_(+)
MYCN_(+)
ZNF318_(+)
ETV4_(+)
MXD3_(+)
TBX10_(+)
FOXM1_(+)
E2F7_(+)
BRCA1_(+)
SOX18_(+)
ZEB1_(+)
NFIB_(+)
RARG_(+)
ERG_(+)
Regulon
I
HALLMARK_EPITHELIAL_MESENCHYMAL_TRANSITION
Running Enrichment Score
NES=2.88
p=1e-04
J
HALLMARK_BILE_ACID_METABOLISM
Running Enrichment Score
NES=1.32
p=9e-04

Supplementary Figure S3 Genomic and transcriptomic features of HB(A-B) Bar plots showing mutated genes in HB PT and AT samples categorized by variant classification, variant type, and SNV classes. **(C-D)** Bar chart showing the enrichment of GO and KEGG terms based on mutated genes in HB PT and AT samples.**(E)** Heatmap showing the inferred CNV profiles in malignant cells of HB and their comparison with non-malignant cells.**(F)** Bar chart showing the enrichment of GO and KEGG terms based on signature genes in each HB tumor subtype.**(G)** UMAP visualization of 3,085 tumor cells from HB based on Scenic regulon AUC scores.**(H)** Rank for regulons in each HB tumor subtype based on regulon specificity score (RSS).**(I)** GSEA enrichment plot of expression signatures of HALLMARK_EPITHELIAL_ MESENCHYMAL_TRANSITION in HB PT malignant cells.**(J)** GSEA enrichment plot of expression signatures of HALLMARK_BILE_ACID_METABOLISM in HB AT malignant cells.

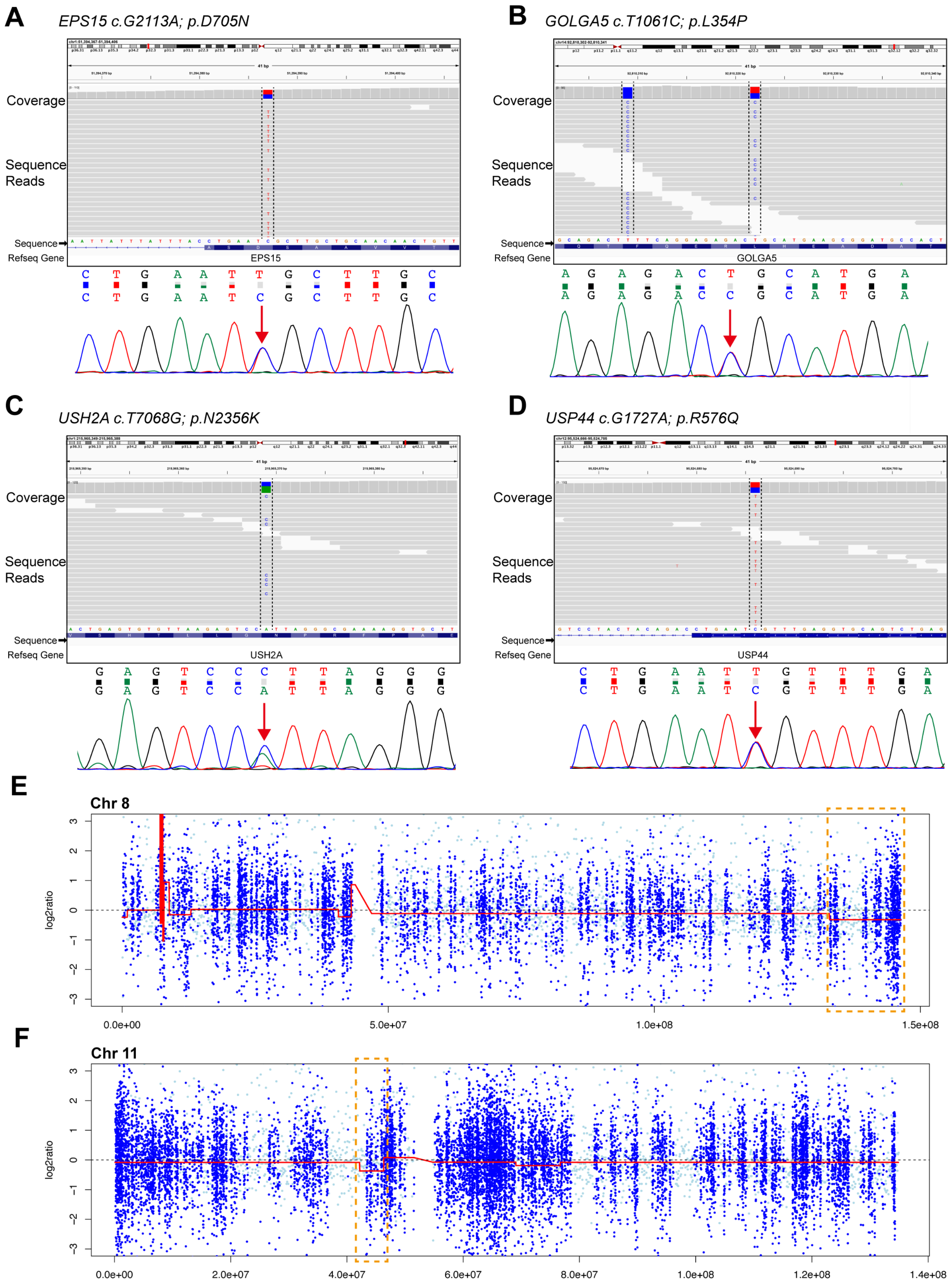
A
EPS15 c.G2113A; p.D705N
Coverage
Sequence Reads
Sequence
Refseq Gene
EPS15
B
GOLGA5 c.T1061C; p.L354P
Coverage
Sequence Reads
Sequence
Refseq Gene
GOLGA5
C
USH2A c.T7068G; p.N2356K
Coverage
Sequence Reads
Sequence
Refseq Gene
USH2A
D
USP44 c.G1727A; p.R576Q
Coverage
Sequence Reads
Sequence
Refseq Gene
USP44
E
Chr 8
log2ratio
0.0e+00
5.0e+07
1.0e+08
1.5e+08
F
Chr 11
log2ratio
0.0e+00
2.0e+07
4.0e+07
6.0e+07
8.0e+07
1.0e+08
1.2e+08
1.4e+08

Supplementary Figure S4 Germline SNPs and CNVs of the patient(A-D) IGV screenshot (upper) and DNA sequence chromatogram of Sanger sequencing (bottom) of potential pathogenic germline mutations. **(E)** Scatter plots showing germline copy number alterations in chromosome 8. **(F)** Scatter plots showing germline copy number alterations in chromosome 11.
